# Protective Immunity against SARS Subunit Vaccine Candidates Based on Spike Protein: Lessons for Coronavirus Vaccine Development

**DOI:** 10.1155/2020/7201752

**Published:** 2020-07-18

**Authors:** Atin Khalaj-Hedayati

**Affiliations:** School of Biosciences, Faculty of Health and Medical Sciences, Taylor's University, Subang Jaya, Malaysia

## Abstract

The recent outbreak of the novel coronavirus disease, COVID-19, has highlighted the threat that highly pathogenic coronaviruses have on global health security and the imminent need to design an effective vaccine for prevention purposes. Although several attempts have been made to develop vaccines against human coronavirus infections since the emergence of Severe Acute Respiratory Syndrome coronavirus (SARS-CoV) in 2003, there is no available licensed vaccine yet. A better understanding of previous coronavirus vaccine studies may help to design a vaccine for the newly emerged virus, SARS-CoV-2, that may also cover other pathogenic coronaviruses as a potentially universal vaccine. In general, coronavirus spike protein is the major antigen for the vaccine design as it can induce neutralizing antibodies and protective immunity. By considering the high genetic similarity between SARS-CoV and SARS-CoV-2, here, protective immunity against SARS-CoV spike subunit vaccine candidates in animal models has been reviewed to gain advances that can facilitate coronavirus vaccine development in the near future.

## 1. Introduction

Before 2003, coronaviruses were known to cause only common cold in humans, but currently, they are the reason for three outbreaks in the 21^st^ century: 2003 Severe Acute Respiratory Syndrome (SARS), 2012 Middle East Respiratory Syndrome (MERS), and 2019 Coronavirus Disease (COVID-19) [1]. Newly emerged virus, named SARS-CoV-2, was first discovered in December 2019, and in a short span of time, it has been announced as a global pandemic. On 2 June 2020, the virus spread has been noted in 213 countries and total confirmed cases climbed above 6.1 million with over 376000 deaths [2]. Despite numerous attempts to develop a vaccine against human coronavirus infection, there is no commercial vaccine available yet. Safety considerations and the degree of extensive diversity in antigenic variants are some of the potential reasons that limit coronavirus vaccine development [3].

Coronaviruses are enveloped positive-sense RNA virus, which are host-specific and can infect the human and a large number of animals [4]. Nucleotide substitution has been proposed to be one of the most important mechanisms of viral evolution in nature, and it is not necessarily surprising for an RNA virus that is a measurably evolving population over a short time, to have distinct variants [5, 6]. Coronaviruses are phylogenetically classified into four major genera: *Alphacoronavirus*, *Betacoronavirus*, *Gammacoronavirus*, and *Deltacoronavirus*, and all three highly pathogenic coronaviruses for human belong to the *Betacoronavirus* genus. All epidemiological, pathophysiological, and immunological researches, which have been done on *Betacoronaviruses* may shed light on the understanding of SARS-CoV-2. This newly emerged virus is genetically more closer to SARS-CoV than MERS coronavirus with the existence of 380 amino acid substitution differences in the encoded proteins [7–9]. Therefore, previous advances made in developing SARS-CoV vaccines could be exploited for designing a vaccine not only for current COVID-19 pandemic but also for other highly pathogenic coronaviruses, so-called universal vaccine. This vaccine can be effective against all strains of the virus as a consequence of cross-protective immunity against conserved antigens. Moreover, the induced broad immunity can prevent the human from infection in the time of emerging a novel strain of the virus.

Inactivated virus and subunit vaccine technologies have been used to develop SARS-CoV vaccines. The inactive virus strategy is limited by safety considerations, as large quantities of the pathogenic virus are required directly in the vaccine preparation procedures. In contrast, the subunit vaccine that only relies on the antigen of interest by using recombinant technology is considered as a more reliable and safe technique. However, low immunogenicity might be a drawback in subunit vaccine development due to poor presentation to the immune system or incorrect folding of the antigens, but adjuvants can be involved in vaccination to boost immune responses and increase immunogenicity [10]. Alternatively, knowledge of the various viral proteins in inducing immune responses would facilitate subunit vaccine preparations [11]. The genome of coronaviruses includes a variable number of open reading frames that encode accessory proteins, nonstructural proteins, and structural proteins [12]. Most of the antigenic peptides are located in the structural proteins [13]. Spike surface glycoprotein (S), a small envelope protein (E), matrix protein (M), and nucleocapsid protein (N) are four main structural proteins. Since S-protein contributes to cell tropism and virus entry and also it is capable to induce neutralizing antibodies (NAb) and protective immunity, it is recognized as the most important target in coronavirus vaccine development among all other structural proteins [3, 14–17]. Moreover, amino acid sequence analysis has shown that S-protein contains conserved regions among the coronaviruses, which may be the basis for universal vaccine development [18, 19]. This article reviewed the in vivo protective immunity of SARS-CoV S-protein vaccine candidates to provide an immunological evidence base that can aid coronavirus vaccine development in the future.

## 2. Search Strategy and Selection Criteria

References for this review were identified through searches of Scopus and PubMed for articles published between 2003 and 2020, using combinations of the terms “Severe Acute Respiratory Syndrome,” SARS, SARS-CoV, vaccin^∗^, immuniz^∗^, immunis^∗^, innocul^∗^, develop^∗^, design^∗^, immunogenicity, and “immune response.” The articles that indicated in vivo protective immunity study have been selected from the search result. The final reference list was generated based on this selection and relevant articles to the subtopics covered in this review, to highlight the immunological evidence base for coronavirus vaccine development and provide recommendations for navigating the limitations.

## 3. Coronavirus Spike Protein: Promising Immunogen for Universal Vaccine Development

**Figure 1:**
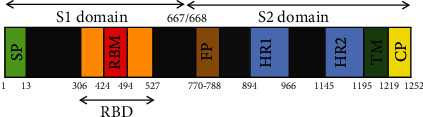
Schematic structure of the spike protein. Abbreviations: SP: signal peptide; RBD: receptor-binding domain; RMB: receptor motif binding; FP: fusion peptide; HR1: heptad repeat domain 1; HR2: heptad repeat domain 2; TM: transmembrane domain; CP: cytoplasmic domain. Numbers indicate amino acid sequence.

The trimetric S-protein of coronaviruses is the reason for the crown shape of the viral particles, from which the name of the virus was given [20]. This structural protein belongs to class I viral fusion proteins and plays essential roles in the cell receptor binding, host tissue tropism and, pathogenesis. S-protein is cleaved at the S1/S2 site by host cell proteases during infection. Following cleavage, the protein is divided into an S1-ectodomain that recognizes a cognate cell surface receptor and an S2-membrane-anchored protein involved in viral entry [21]. The S1-protein contains a receptor-binding domain (RBD), which recognizes the angiotensin-converting enzyme 2 (ACE2) as one of the main receptors in the host [22]. RBD contains receptor-binding motif (RBM) that makes all the contacts with ACE2 and RBD core that is mostly conserved among coronaviruses [21, 23]. Binding of the RBD to the ACE2 receptor provokes S2 conformational changes [24]. S2 contains membrane-anchoring, fusion peptide, and two heptad repeat domains (HR1 and HR2), which plays a key role in virus assembly and entry (Figure 1) [25–27]. The studies showed that vaccines based on S-protein can evoke the immune system to induce humoral and cellular responses and protect vaccinated animals from SARS viral challenges (Table 1) [17, 28–32].

In general, vaccine development for pathogens with a high rate of antigenic changes in their surface antigens such as SARS-CoV is difficult and complicated due to the emergence of vaccine escape variants over time. Hence, identification of conserved antigenic determinant on S-protein, which can induce cross-protective immune responses, may have implications in the development of an effective vaccine against all pathogenic coronaviruses. There is a limited number of SARS vaccine studies based on conserved antigens in which the potential protective immunity was not evaluated. For example, Zhang et al. identified a highly conserved antigenic determinant on S2 (amino acid 803 to 828) by using sera from convalescent SARS patients that could induce the S2-specific antibody with in vitro neutralizing activity against the SARS-CoV pseudovirus [33]. Other laboratory results indicated that peptides from the HR2 region of the S2 domain can block SARS-CoV infection in vitro [34, 35]. However, the potential protection was not evaluated by challenge experiments, and further investigation is required to check in vivo neutralizing activity and also cross-protective immunity of these antigens for broad immune responses. Moreover, recent genome analysis disclosed that S2 and RBD core domains of SARS-CoV-2 are highly conserved, supporting the idea of universal vaccine design [8, 9, 18, 36–38]. It is notable that functional sites of S2 domain might be buried under S1 in the native state of the virus structure, and it may affect the accessibility of the S2 antigens for the immune system but despite this shielding effect, T cell immune response to S2 domain that has been reported from fully recovered SARS patients suggests that it can be a candidate antigen for coronavirus vaccine development [39]. In addition, extra caution should be taken in designing vaccines that specifically target the S2 domain as disease severity can be augmented by elevating viral fusion to host cells at the early stage of the infection before activation of prior vaccine-induced immune responses [40, 41].

## 4. What Immune Responses Are Required after Coronavirus Vaccination?

Based on clinical studies, we can understand how the immune system of the patients reacts to coronavirus [42, 43]. In general, the viral infection will be responded with humoral and cellular responses, which will be initiated by the innate immune system followed by the adaptive immune response. The latter consists of B cells that produce antibodies and T cells that kill virus-infected cells and both induce memory responses [44]. Generally, NAbs are the key factor for protecting human and animal models from coronavirus infections [17, 28, 45]. Sera of SARS patients indicate the presence of antibodies against SARS-CoV S, M, E, and N proteins, but NAb is only induced by S-protein [46]. Although anti-N antibody is present in SARS patient sera at a high-level and persisted for a long time (30 weeks after infection), recombinant N-protein could not induce a detectable NAb in rabbit to neutralize the SARS-CoV infection [11]. Besides, deficient antibody production against S-protein in SARS-CoV infected patients with fatal outcomes has been reported that emphasizes the crucial role of the S-protein NAb in SARS immunity [47]. Antibodies from the sera of 623 SARS patients were able to neutralize viruses containing S-protein from four different SARS-CoV strains, suggesting the potential cross-reactivity of these antibodies [48]. SARS-CoV-specific NAbs peak at four months and after that, gradually decline over time, indicating a reduction of the memory B cells against the virus [46, 49–51].

On the other hand, memory CD8 T cells can persist for at least six years in SARS patients who had recovered from the infection. These T cells are able to recognize and remove the infected cells in the lungs of patients. Whereas the memory B cell response and consequently NAbs are short-lived in SARS patients, generating long-lived memory T cell response is important, and it can be a complementary strategy in SARS vaccine design [46]. Interestingly, among the SARS-CoV CD8 T cell epitopes derived from different structural proteins, most peptides belong to S-protein [52]. In a clinical phase I study, a truncated S-protein DNA vaccine produced in bacterial cells induced SARS S-protein specific T cell response in all subjects and NAb responses detected in 80% of the individuals [53]. A DNA S-protein vaccine induced both CD4 and CD8 T cell responses, and S2 fragment encoding amino acid 681-980 elicited specific Cytotoxic T lymphocyte (CTL) response in animal models [41, 54]. In another preclinical study, S-protein specific memory CD8 T cells could protect the mice against viral lethal challenge in the absence of NAbs, indicating that protection was elicited by memory CD8 T cells [55]. Also, dysregulated innate immune response that is a critical factor in the pathogenesis of SARS-CoV can be negated by a potent T cell response [56]. Subsequently, the induction of cellular immune response is a goal for vaccine preparations as it plays an important role in antiviral immunity [53, 55, 57, 58].

Moreover, the studies suggested that mucosal immune responses represented by secretory IgA will also be important in the prevention of SARS-CoV infection [45]. Hence, intranasal (IN) immunization may be a preferable route to generate lung memory T cells and IgA specifically. However, IN immunization that produced local immunity in the upper respiratory tract but not in the lower tract where the virus replicates, performed less lung virus titer reduction than intramuscular (IM) route [59]. So, mucosal immune response localization should be considered while designing IN coronavirus vaccines. Eventually, future vaccines against coronaviruses should emphasize the generation of systematic and mucosal memory T cell responses and induction of long-lived NAb against S-protein for optimal protection and virus clearance in the time of infection. Further, cross-protective immunity needs to be considered in the aim of coronavirus universal vaccine design.

## 5. In Vivo Protective Immunity of SARS-CoV Spike-Based Vaccine Candidates

In this article, recombinant S-protein-based vaccines, which include full-length or fragment vaccines, DNA-based vaccine, and viral vector-based vaccines that induce protective immunity in animal models have been reviewed (Table 1).

### 5.1. Protein-Based Vaccine

Full-length S-protein vaccines might be able to induce unwanted immune responses resulting in antibody-mediated disease enhancement (ADE) that can cause inflammatory and liver damage or enhancing infection after being challenged with SARS-CoV in animal models [60–63]. On the other hand, it has been demonstrated that fragments of S-protein, such as truncated S-protein or RBD, have a great potential to be effective vaccine candidates against SARS with no evidence of harmful immune responses [28, 64, 65]. In several studies, Du and colleagues proposed SARS-CoV RBD as a fragment immunogen that can induce high titers of NAb and protective immunity in mice without immunopathological damages after being a challenge with the virus [17, 66, 67]. Administration of SARS-CoV recombinant S polypeptide (amino acids 14 to 762) elicited NAb in mice and protected the animals against upper and lower respiratory infection with SARS-CoV. This protein segment contains the RBD region as well as immunodominant and neutralizing epitopes [31, 68, 69].

To obtain highly expressed RBD economically and conveniently with biological functions, 193 amino acids of RBD (RBD-193) residues 318–510, have been expressed in different systems: mammalian cells 293T (RBD193-293T) and chinese hamster ovary K1 (RBD193-CHO-K1), insect cells Sf9 (RBD193-Sf9) and, *E. coli* (RBD193- Ec) [28, 70]. All RBD expressed proteins except RBD193-CHO-K1 induced complete protection against viral challenge in immunized mice. Virus replication was detected in two of five mice which were vaccinated with RBD193-CHO-K1. Interestingly, by adding 26 amino acids at the C terminal of RBD193 while expressed in the same CHO-K1 cell line (RBD219-CHO-K1), full protection against SARS-CoV challenge was observed due to high titers of RBD-specific antibody production. The extension may affect RBD structure into more immunogenic or more stable conformation, which could cause the induction of more potent protective antibodies in animal models compared to RBD193-CHO-K1. Cellular immune responses were also detected in RBD193-CHO-K1 and RBD219-CHO-K1 vaccinated mice [28, 70]. Although the mammalian expressed RBD elicited stronger NAb responses than those expressed in insect and *E. coli*, Du and colleagues suggested any immunogens that elicited the serum NAb titers of >1 : 500 in vaccinated mice would be effective enough for protection. So RBD193-Sf9 and RBD193-Ec were also considered as effective vaccines against viral challenge in mice with mean value >1 : 700 for the serum NAb titers [28]. In general, insect and *E. coli* expression systems have lower production costs with a high productivity rate compared to the mammalian cell expression system, which make them more feasible for mass vaccine production [71, 72].

As the subunit vaccine may represent low immune responses, adjuvants or immunopotentiator can be used to increase immunogenicity by helping better presenting of immunogens to immune cells. The recombinant virus-like particles (VLP) have been shown to be an effective immunopotentiator and delivery system for foreign antigens in vaccine development. The repetitive antigen pattern on their surface can cause a stronger and broader induction of immune responses to the foreign antigen that is incorporated into the VLP [73, 74]. Liu et al. developed SARS S-protein VLP vaccine candidate with similar morphology and size to the SARS-CoV, and the IM immunization of the vaccine protected 100% of vaccinated mice from death and significantly reduced lung virus titer [59]. It is also important to concern about structural changes of immunogens that may reduce the immunogenicity after adjuvant implications in vaccine development. In one study, gold nanoparticles (AuNPs) containing S-protein showed weak protective ability against the SARS-CoV challenge compared to the control group because of the low induction of virus-specific IgG and NAb, which may be related to S-protein structural changes upon binding to AuNPs [63].

In the case of respiratory infections like SARS, both serum and lung immune responses are important, and it has been shown that relying on a systemic response may not be enough to reduce SARS-CoV infection in host [55, 75]. Mucosal immunization offers several advantages over other routes of antigen delivery, including convenience, cost-effectiveness, and induction of both local and systemic immune responses [64, 76]. One study indicated that only IN immunization of mice by truncated S-protein with protollin adjuvant induced antigen-specific IgA responses in lung lavage fluid while both IN and IM administrations of the vaccine elicited comparable systemic responses. Serum collected IgG was able to possess strong protective activity against SARS-CoV, but the virus titer in the lung of mice was much lower for the IN vaccine compared to the IM route, and there was a qualitative correlation between the level of IgA and virus titer in the lung of animals. The fact that IM immunization developed a high level of serum antibodies but not detectable mucosal IgA with higher viral lung titer than IN route strongly indicates the essential role of the mucosal responses in SARS immunity [77].

### 5.2. DNA and Viral Vector-Based Vaccines

Numbers of DNA and viral vectored vaccines against SARS-CoV have been explored. A DNA vaccine that encodes full-length SARS S-protein induced NAb and T cell responses and stimulated protective immunity [78]. Also, S-protein-based DNA vaccine encoding posttranscriptional enhancer (pCI-WPRE-S) not only improved immunogenicity in mice but also lessen DNA vaccine amount in vaccination. 10 *μ*g of the vaccine elicited NAb response equivalent to 25 *μ*g of Yang et al. codon-optimized vector [78, 79]. The vaccine even at a dose as low as 2 *μ*g protects immunized animal against challenge infection [79]. This modification of DNA vaccine can minimize the risk of autoimmune responses or integration of the foreign DNA into the host genome [80]. The DNA S-protein vaccine was able to elicit SARS-CoV-specific CD4 T cell responses in all tested individuals and CD8 T cell responses in 20% of subjects. Also, in prior clinical trials of DNA vaccines against HIV, Ebola, and West Nile virus, vaccine-specific CD4 T cell responses were detected in nearly all subjects, while the frequency of measurable CD8 T cell responses varied from 7% to 64% [53, 81–83]. This aspect of DNA vaccine-induced immunity will require additional considerations while designing a DNA vaccine for coronaviruses.

The live viral vectored vaccines do not involve the complete pathogen, and they are qualified to induce mucosal humoral immune responses that may not be easily happened by DNA or protein vaccines [84, 85]. A vesicular stomatitis virus (VSV) and an attenuated version of the human parainfluenza virus, a common respiratory pathogen in humans, both expressing the SARS-CoV S protein, were protective in animal models against SARS-CoV [86, 87]. However, any viral vector vaccine based on common pathogens in the population would bring the concern regarding significant existence of prior immunity, which may restrict the replication of the viral vector in immunized models and decline the immunogenicity [88, 89]. Newcastle disease virus (NDV) is another viral vector that is antigenically distinct from a common human pathogen, and its natural host is birds. Remarkably, inoculation of the respiratory tract of African green monkeys (AGM) with recombinant NDV encoding SARS S-protein was protective enough against a high challenge dose of SARS-CoV. Vaccination with two constructs of NDV (NDV-VF/S or NDV-BC/S) resulted in a titer of serum NAb that was equaled or exceeded to parainfluenza construct immunization results [87, 90]. Mice immunized with modified vaccinia virus Ankara that contains S-protein (MVA-S) developed NAb and exhibited little or no replication of SARS CoV in the upper and lower respiratory tracts with no obvious disease after inoculation [91]. However, NAb responses in ferret against MVA-S were not protective in animals, but produce strong ADE effects [60, 61]. As S-protein DNA-based vaccine, MVA-S, and a live SARS-CoV demonstrated significant protective immunity in mice, different replication kinetics for SARS-CoV in mice and ferret can be the most likely cause for the difference in protective efficacy in these two animals [61, 78, 91, 92]. Thus, extra caution should be taken in the proposed human trials of SARS vaccines due to the potential liver damage from immunization and virus infection.

The IN vaccination with adeno-associated virus (AAV) encoding SARS-RBD induced strong systemic humoral (IgG) and pulmonary humoral (IgA) responses with neutralizing activity as compared to IM vaccination. The immunization also protects BALB/c mice against SARS-CoV infection. Surprisingly, AAV-RBD vaccination induced stronger CTL responses both in the lung and the spleen, and no sign of ADE in the animals was observed. Besides low systematic antibody responses in IN vaccination compared to the IM route, higher protection against virus challenge was achieved. The protective efficacy of AAV-RBD vaccination against SARS-CoV infection is correlated with the antibodies level, especially lung IgA [45]. Therefore, compared with the IM route, IN vaccination may fulfill multiple criteria for an effective and safe SARS-CoV vaccine.

## 6. Conclusion

The development of effective SARS-CoV-2 vaccine is crucial in aid of our public health preparedness against the current COVID-19 pandemic. Improved understanding of the protective immunity of SARS S-protein vaccine candidates would provide the immunological evidence base for future vaccine production. Moreover, this review reveals that despite the evidence of cross-reactivity of spike protein antibodies from SARS patients, there is a profound gap for immune response cross-protective evaluation against different strains of the virus. Now, it is time to direct research toward universal vaccine development by focusing on conserved immunogens that elicit potential cross-protective immunity to reduce the global threat of SARS-CoV-2 and any other pathogenic coronaviruses. Such studies would include preclinical experiments and also early phase clinical trials to assess the vaccine's immunogenicity. On the other hand, the previous vaccine studies of coronavirus have shown that immunization could often lead to adverse effect, such as ADE in animal models. Thus, it is important to clearly understand the potential risks of coronavirus vaccines and extra caution must be taken while testing the designed vaccines in human trials. In general, optimization of the vaccination regimen and evaluation of different vaccine strategies may be helpful to improve vaccine safety.

## Figures and Tables

**Table 1:** In vivo immunological responses against SARS-CoV spike-based vaccine candidates.

Vaccine type	Vaccine compound	Production system	Adjuvant	Route	Animal model	Immune response			Side effect	Note	Reference
						Humoral	cellular	protective immunity			
Protein based	S-protein Residues 14-762	Insect cells	Saponin: QS21	S.C.	Mice	✓	✓	✓	N/A	The better significant protection obtained with the QS21.	[31]
			Ribi: MPL+TDM			✓	✓	✓			
	RBD-Fc (193 aa) Residues 318- 510	Mammalian 293T cells	Freund's complete adjuvant. Boost: Freund's incomplete adjuvant	I.M.	Mice	✓	N/A	✓	Only one vaccinated mouse from five had mild alveolar damage in the lung tissues.		[17]
	RBD (193 aa)	Mammalian 293T cells	Sigma adjuvant system	S.C.	Mice	✓	N/A	✓	N/A		[28]
	Residues 318–510	Insect cells				✓	N/A	✓			
		*E. coli*				✓	N/A	✓			
	RBD (193 aa) Residues 318–510	(CHO)-K1 cells	Freund's complete Boost: Freund's in-complete	S.C.	Mice	✓	✓	✓	N/A	Virus replication detected in two of five mice.	[70]
	RBD (219 aa) Residues 318–536	(CHO)-K1 cells	Freund's complete Boost: Freund's in-complete	S.C.	Mice	✓	✓	✓	N/A		[66]
	Truncated S-protein	Insect cells	Protollin	I.N.	Mice	✓	N/A	✓	No significant histopathology findings	Significantly lower virus titers in I.N. route than I.M due to induction of IgA	[77]
			Alum	I.M.		✓	N/A	✓			
	SARS-VLPs	Insect cells	Without adjuvant	I.M.	Mice	✓	N/A	✓	No weight loss.		[59]
	S-protein Residues 1-1196aa + Influenza M1 protein Residues 531-568aa		Without adjuvant	I.N.		✓	N/A	✓	Minor weight loss (3-4%)		
			Alum	I.M.		✓	N/A	✓	No weight loss		
	S-protein Residues 1-1196aa	Insect cells	Without adjuvant	I.M.	Mice	✓	N/A	✓ 70% survival^∗^	7.5-15% Weight loss		[59]
			Without adjuvant	I.N.		✓	N/A	x			
			Alum	I.M.		✓	N/A	✓	No weight loss		
	S-protein Residues 1–1194	Insect cells	AuNPs	S.C.	Mice	✓	N/A	✓ 43% survival^∗^	Ruffled fur and body weight loss for all groups. Eosinophilic infiltrations in the lungs of both S-protein and S +AuNP immunized mice		[63]
			TLR agonists			✓	N/A	✓			
			Without adjuvant			✓	N/A	✓ 66% survival^∗^			

Viral vector based	MVA-S Full length	Primary CEF cells	Without adjuvant	I.M. I.N.	Mice	✓ ✓	N/A N/A	✓ ✓	No obvious disease		[91]
	MVA-S Full length	BHK21 cells	Without adjuvant	S.C. and I.P.	Ferrets	✓	N/A	X	Enhanced hepatitis in ferrets after SARS-CoV challenge	No significant effects on the level of SARS-CoV replication in ferrets.	[61]
	MVA-S Full length	BHK21 cells	Without adjuvant	S.C. and I.P.	Ferrets	✓	N/A	X	No enhanced pathology during SARS-CoV infection of liver	Presence of virus in all the clinical specimens.	[60]
	MVA-N Full length					X	N/A	X			
	Attenuated parainfluenza virus (BHPIV3)-S protein Full length	LLC-MK2 cells	Without adjuvant	I.N.	African green monkeys	✓	N/A	✓	No evidence of immune-mediated enhancement of infection or disease		[87]
	NDV-BC/S Full-length	Embryonated chicken eggs	Without adjuvant	I.N. and I.T.	African green monkeys	✓	✓	✓	No evidence of enhanced clinical disease	Numbers of blood SARS-CoV specific T-cells was low	[90]
	NDV-VF/S Full-length NDV-BC/S1 Residues 1-762					✓	✓	✓			
						N/A	N/A	X			
	AAV-RBD (193-aa)	HEK293T cells	Without adjuvant	I.M.	Mice	✓	✓	✓	No antibody-mediated disease enhancement	I.N. vaccination induced much stronger responses than I.M. route but with the same protective immunity effectiveness.	[45]
	Residue 318–510			I.N.		✓	✓	✓			
	VSV-S Full length	BHK-21 cells	Without adjuvant	I.N.	Mice	✓	N/A	✓	No enhancement of infection		[86]

DNA based	pCI-WPRE-S Full length	Mammalian 293T cells	Without adjuvant	I.M.	Mice	✓	N/A	✓	N/A		[79]
	Plasmid DNA-S Full length	Synthetic Human preferred codons	Without adjuvant	I.M.	Mice	✓	✓	✓	No enhancement of infection	Protection was mediated by a humoral immune mechanism.	[78]

Abbreviations: I.M.: intramuscular; I.N.: intranasal; S.C.: subcutaneous; I.P.: intraperitoneal; I.T.: intrathecal; N/A: not available; X: not induced. ^∗^Survival rate is given under the protective immunity column if it was indicated in the reference article.

## References

[1] Reed S. E. (1984). The behavior of recent isolates of human respiratory coronavirus in vitro and in volunteers: evidence of heterogeneity among 229E-related strains. *Journal of Medical Virology*.

[2] Coronavirus disease 2019 (2020). *World Heal Organ*.

[3] Graham R. L., Donaldson E. F., Baric R. S. (2013). A decade after SARS: strategies for controlling emerging coronaviruses. *Nature Reviews Microbiology*.

[4] Weiss S. R., Navas-Martin S. (2005). Coronavirus pathogenesis and the emerging pathogen severe acute respiratory syndrome coronavirus. *Microbiology and Molecular Biology Reviews*.

[5] Lauring A. S., Andino R. (2010). Quasispecies theory and the behavior of RNA viruses. *PLoS Pathogens*.

[6] Xu D., Zhang Z., Wang F. S. (2004). SARS-associated coronavirus quasispecies in individual patients. *The New England Journal of Medicine*.

[7] Fung T. S., Liu D. X. (2019). Human coronavirus: host-pathogen interaction. *Annual Review of Microbiology*.

[8] Zhang Y.-Z., Holmes E. C. (2020). A genomic perspective on the origin and emergence of SARS-CoV-2. *Cell*.

[9] Wu A., Peng Y., Huang B. (2020). Genome composition and divergence of the novel coronavirus (2019-nCoV) originating in China. *Cell Host & Microbe*.

[10] Purcell A. W., McCluskey J., Rossjohn J. (2007). More than one reason to rethink the use of peptides in vaccine design. *Nature Reviews Drug Discovery*.

[11] Qiu M., Shi Y., Guo Z. (2005). Antibody responses to individual proteins of SARS coronavirus and their neutralization activities. *Microbes and Infection*.

[12] Song Z., Xu Y., Bao L. (2019). From SARS to MERS, thrusting coronaviruses into the spotlight. *Viruses*.

[13] Cui J., Li F., Shi Z. L. (2019). Origin and evolution of pathogenic coronaviruses. *Nature Reviews Microbiology*.

[14] Li C. K., Wu H., Yan H. (2008). T cell responses to whole SARS coronavirus in humans. *Journal of Immunology*.

[15] He Y., Li J., Heck S., Lustigman S., Jiang S. (2006). Antigenic and immunogenic characterization of recombinant baculovirus-expressed severe acute respiratory syndrome coronavirus spike protein: implication for vaccine design. *Journal of Virology*.

[16] Simmons G., Reeves J. D., Rennekamp A. J., Amberg S. M., Piefer A. J., Bates P. (2004). Characterization of severe acute respiratory syndrome-associated coronavirus (SARS-CoV) spike glycoprotein-mediated viral entry. *Proceedings of the National Academy of Sciences of the United States of America*.

[17] Du L., Zhao G., He Y. (2007). Receptor-binding domain of SARS-CoV spike protein induces long-term protective immunity in an animal model. *Vaccine*.

[18] Wan Y., Shang J., Graham R., Baric R. S., Li F. (2020). Receptor recognition by the novel coronavirus from Wuhan: an analysis based on decade-long structural studies of SARS Coronavirus. *Journal of Virology*.

[19] Rota P. A., Oberste M. S., Monroe S. S. (2003). Characterization of a novel coronavirus associated with severe acute respiratory syndrome. *Science*.

[20] Marra M. A., Jones S. J. M., Astell C. R. (2003). The genome sequence of the SARS-associated coronavirus. *Science*.

[21] Li F. (2016). Structure, function, and evolution of coronavirus spike proteins. *Annual Review of Virology*.

[22] Li W., Moore M. J., Vasilieva N. (2003). Angiotensin-converting enzyme 2 is a functional receptor for the SARS coronavirus. *Nature*.

[23] Li F., Li W., Farzan M., Harrison S. C. (2005). Structure of SARS coronavirus spike receptor-binding domain complexed with receptor. *Science*.

[24] Li F., Berardi M., Li W., Farzan M., Dormitzer P. R., Harrison S. C. (2006). Conformational states of the severe acute respiratory syndrome coronavirus spike protein ectodomain. *Journal of Virology*.

[25] Gallagher T. M., Buchmeier M. J. (2001). Coronavirus spike proteins in viral entry and pathogenesis. *Virology*.

[26] Holmes K. V. (2003). SARS-associated coronavirus. *The New England Journal of Medicine*.

[27] Sainz B., Rausch J. M., Gallaher W. R., Garry R. F., Wimley W. C. (2005). Identification and characterization of the putative fusion peptide of the severe acute respiratory syndrome-associated coronavirus spike protein. *Journal of Virology*.

[28] Du L., Zhao G., Chan C. C. S. (2009). Recombinant receptor-binding domain of SARS-CoV spike protein expressed in mammalian, insect and *E. coli* cells elicits potent neutralizing antibody and protective immunity. *Virology*.

[29] Hu H., Lu X., Tao L. (2007). Induction of specific immune responses by severe acute respiratory syndrome coronavirus spike DNA vaccine with or without interleukin-2 immunization using different vaccination routes in mice. *Clinical and Vaccine Immunology*.

[30] Chen Z., Zhang L., Qin C. (2005). Recombinant modified vaccinia virus Ankara expressing the spike glycoprotein of severe acute respiratory syndrome coronavirus induces protective neutralizing antibodies primarily targeting the receptor binding region. *Journal of Virology*.

[31] Bisht H., Roberts A., Vogel L., Subbarao K., Moss B. (2005). Neutralizing antibody and protective immunity to SARS coronavirus infection of mice induced by a soluble recombinant polypeptide containing an N-terminal segment of the spike glycoprotein. *Virology*.

[32] Zhou T., Wang H., Luo D. (2004). An exposed domain in the severe acute respiratory syndrome coronavirus spike protein induces neutralizing antibodies. *Journal of Virology*.

[33] Zhang H., Wang G., Li J. (2004). Identification of an antigenic determinant on the S2 domain of the severe acute respiratory syndrome coronavirus spike glycoprotein capable of inducing neutralizing antibodies. *Journal of Virology*.

[34] Zhu J., Xiao G., Xu Y. (2004). Following the rule: formation of the 6-helix bundle of the fusion core from severe acute respiratory syndrome coronavirus spike protein and identification of potent peptide inhibitors. *Biochemical and Biophysical Research Communications*.

[35] Keng C.-T., Zhang A., Shen S. (2005). Amino acids 1055 to 1192 in the S2 region of severe acute respiratory syndrome coronavirus S protein induce neutralizing antibodies: implications for the development of vaccines and antiviral agents. *Journal of Virology*.

[36] Walls A. C., Park Y. J., Tortorici M. A., Wall A., McGuire A. T., Veesler D. (2020). Structure, function, and antigenicity of the SARS-CoV-2 spike glycoprotein. *Cell*.

[37] Chan J. F.-W., Kok K. H., Zhu Z. (2020). Genomic characterization of the 2019 novel human-pathogenic coronavirus isolated from a patient with atypical pneumonia after visiting Wuhan. *Emerging Microbes & Infections*.

[38] Malik Y. S., Sircar S., Bhat S. (2020). Emerging novel coronavirus (2019-nCoV)—current scenario, evolutionary perspective based on genome analysis and recent developments. *Veterinary Quarterly*.

[39] Wang Y.-D., Sin W. Y. F., Xu G. B. (2004). T-cell epitopes in severe acute respiratory syndrome (SARS) coronavirus spike protein elicit a specific T-Cell immune response in patients who recover from SARS. *Journal of Virology*.

[40] Khurana S., Loving C. L., Manischewitz J. (2013). Vaccine-induced anti-HA2 antibodies promote virus fusion and enhance influenza virus respiratory disease. *Science Translational Medicine*.

[41] Guo Y., Sun S., Wang K., Zhang S., Zhu W., Chen Z. (2005). Elicitation of immunity in mice after immunization with the S2 subunit of the severe acute respiratory syndrome coronavirus. *DNA and Cell Biology*.

[42] Li G., Chen X., Xu A. (2003). Profile of specific antibodies to the SARS-associated coronavirus. *The New England Journal of Medicine*.

[43] Corti D., Zhao J., Pedotti M. (2015). Prophylactic and postexposure efficacy of a potent human monoclonal antibody against MERS coronavirus. *Proceedings of the National Academy of Sciences of the United States of America*.

[44] Chaplin D. D. (2010). Overview of the immune response. *Journal of Allergy and Clinical Immunology*.

[45] Du L., Zhao G., Lin Y. (2008). Intranasal vaccination of recombinant adeno-associated virus encoding receptor-binding domain of severe acute respiratory syndrome coronavirus (SARS-CoV) spike protein induces strong mucosal immune responses and provides long-term protection against SARS-CoV infection. *Journal of Immunology*.

[46] Chen J., Subbarao K. (2007). The immunobiology of SARS. *Annual Review of Immunology*.

[47] Cameron M. J., Ran L., Xu L. (2007). Interferon-mediated immunopathological events are associated with atypical innate and adaptive immune responses in patients with severe acute respiratory syndrome. *Journal of Virology*.

[48] Nie Y., Wang G., Shi X. (2004). Neutralizing antibodies in patients with severe acute respiratory syndrome–associated coronavirus infection. *The Journal of Infectious Diseases*.

[49] Woo P. C. Y., Lau S. K. P., Wong B. H. L. (2004). Longitudinal profile of immunoglobulin G (IgG), IgM, and IgA antibodies against the severe acute respiratory syndrome (SARS) coronavirus nucleocapsid protein in patients with pneumonia due to the SARS coronavirus. *Clinical and Diagnostic Laboratory Immunology*.

[50] Wu L. P., Wang N. C., Chang Y. H. (2007). Duration of antibody responses after severe acute respiratory syndrome. *Emerging Infectious Diseases*.

[51] Tang F., Quan Y., Xin Z.-T. (2011). Lack of peripheral memory B cell responses in recovered patients with severe acute respiratory syndrome: a six-year follow-up study. *Journal of Immunology*.

[52] Liu W. J., Zhao M., Liu K. (2017). T-cell immunity of SARS-CoV: implications for vaccine development against MERS-CoV. *Antiviral Research*.

[53] Martin J. E., Louder M. K., Holman L. S. A. (2008). A SARS DNA vaccine induces neutralizing antibody and cellular immune responses in healthy adults in a phase I clinical trial. *Vaccine*.

[54] Huang J., Cao Y., Du J., Bu X., Ma R., Wu C. (2007). Priming with SARS CoV S DNA and boosting with SARS CoV S epitopes specific for CD4+ and CD8+ T cells promote cellular immune responses. *Vaccine*.

[55] Channappanavar R., Fett C., Zhao J., Meyerholz D. K., Perlman S. (2014). Virus-specific memory CD8 T cells provide substantial protection from lethal severe acute respiratory syndrome coronavirus infection. *Journal of Virology*.

[56] Zhao J., Zhao J., Perlman S. (2010). T cell responses are required for protection from clinical disease and for virus clearance in severe acute respiratory syndrome coronavirus-infected mice. *Journal of Virology*.

[57] Chen Y. Z., Liu G., Senju S. (2010). Identification of SARS-COV spike protein-derived and HLA-A2-restricted human CTL epitopes by using a new muramyl dipeptide-derivative adjuvant. *International Journal of Immunopathology and Pharmacology*.

[58] Fu T.-M., Dubey S. A., Mehrotra D. V. (2007). Evaluation of cellular immune responses in subjects chronically infected with HIV type 1. *AIDS Research and Human Retroviruses*.

[59] Liu Y. V., Massare M. J., Barnard D. L. (2011). Chimeric severe acute respiratory syndrome coronavirus (SARS-CoV) S glycoprotein and influenza matrix 1 efficiently form virus-like particles (VLPs) that protect mice against challenge with SARS-CoV. *Vaccine*.

[60] Czub M., Weingartl H., Czub S., He R., Cao J. (2005). Evaluation of modified vaccinia virus Ankara based recombinant SARS vaccine in ferrets. *Vaccine*.

[61] Weingartl H., Czub M., Czub S. (2004). Immunization with modified vaccinia virus Ankara-based recombinant vaccine against severe acute respiratory syndrome is associated with enhanced hepatitis in ferrets. *Journal of Virology*.

[62] Honda-Okubo Y., Barnard D., Ong C. H., Peng B. H., Tseng C. T. K., Petrovsky N. (2015). Severe acute respiratory syndrome-associated coronavirus vaccines formulated with delta inulin adjuvants provide enhanced protection while ameliorating lung eosinophilic immunopathology. *Journal of Virology*.

[63] Sekimukai H., Iwata-Yoshikawa N., Fukushi S. (2019). Gold nanoparticle-adjuvanted S protein induces a strong antigen-specific IgG response against severe acute respiratory syndrome-related coronavirus infection but fails to induce protective antibodies and limit eosinophilic infiltration in lungs. *Microbiology and Immunology*.

[64] Lee J.-S., Poo H., Han D. P. (2006). Mucosal immunization with surface-displayed severe acute respiratory syndrome coronavirus spike protein on Lactobacillus casei induces neutralizing antibodies in mice. *Journal of Virology*.

[65] Zhou Z., Post P., Chubet R. (2006). A recombinant baculovirus-expressed S glycoprotein vaccine elicits high titers of SARS-associated coronavirus (SARS-CoV) neutralizing antibodies in mice. *Vaccine*.

[66] Du L., Zhao G., Chan C. C. S. (2010). A 219-mer CHO-expressing receptor-binding domain of SARS-CoV S protein induces potent immune responses and protective immunity. *Viral Immunology*.

[67] He Y., Lu H., Siddiqui P., Zhou Y., Jiang S. (2005). Receptor-binding domain of severe acute respiratory syndrome coronavirus spike protein contains multiple conformation-dependent epitopes that induce highly potent neutralizing antibodies. *Journal of Immunology*.

[68] Babcock G. J., Esshaki D. J., Thomas W. D., Ambrosino D. M. (2004). Amino acids 270 to 510 of the severe acute respiratory syndrome coronavirus spike protein are required for interaction with receptor. *Journal of Virology*.

[69] He Y., Zhou Y., Wu H. (2004). Identification of immunodominant sites on the spike protein of severe acute respiratory syndrome (SARS) coronavirus: implication for developing SARS diagnostics and vaccines. *Journal of Immunology*.

[70] Du L., Zhao G., Li L. (2009). Antigenicity and immunogenicity of SARS-CoV S protein receptor-binding domain stably expressed in CHO cells. *Biochemical and Biophysical Research Communications*.

[71] Boosani C. S., Sudhakar A. (2006). Cloning, purification, and characterization of a non-collagenous anti- angiogenic protein domain from human *α*1 type IV collagen expressed in *Sf9* cells. *Protein Expression and Purification*.

[72] Saitoh H., Uwada J., Azusa K., Ulrich H. D. (2009). Strategies for the Expression of SUMO-Modified Target Proteins in *Escherichia coli*. *BT - SUMO Protocols*.

[73] Bright R. A., Carter D. M., Daniluk S. (2007). Influenza virus-like particles elicit broader immune responses than whole virion inactivated influenza virus or recombinant hemagglutinin. *Vaccine*.

[74] Song H., Wittman V., Byers A. (2010). In vitro stimulation of human influenza-specific CD8+ T cells by dendritic cells pulsed with an influenza virus-like particle (VLP) vaccine. *Vaccine*.

[75] Ma C., Li Y., Wang L. (2014). Intranasal vaccination with recombinant receptor-binding domain of MERS-CoV spike protein induces much stronger local mucosal immune responses than subcutaneous immunization: implication for designing novel mucosal MERS vaccines. *Vaccine*.

[76] Seegers J. F. M. L. (2002). Lactobacilli as live vaccine delivery vectors: progress and prospects. *Trends in Biotechnology*.

[77] Hu M. C., Jones T., Kenney R. T., Barnard D. L., Burt D. S., Lowell G. H. (2007). Intranasal protollin-formulated recombinant SARS S-protein elicits respiratory and serum neutralizing antibodies and protection in mice. *Vaccine*.

[78] Yang Z. Y., Kong W. P., Huang Y. (2004). A DNA vaccine induces SARS coronavirus neutralization and protective immunity in mice. *Nature*.

[79] Callendret B., Lorin V., Charneau P. (2007). Heterologous viral RNA export elements improve expression of severe acute respiratory syndrome (SARS) coronavirus spike protein and protective efficacy of DNA vaccines against SARS. *Virology*.

[80] Cui Z. (2005). DNA vaccine. *Advances in Genetics*.

[81] Catanzaro A. T., Roederer M., Koup R. A. (2007). Phase I clinical evaluation of a six-plasmid multiclade HIV-1 DNA candidate vaccine. *Vaccine*.

[82] Martin J. E., Sullivan N. J., Enama M. E. (2006). A DNA vaccine for Ebola virus is safe and immunogenic in a phase I clinical trial. *Clinical and Vaccine Immunology*.

[83] Martin J. E., Pierson T. C., Hubka S. (2007). A West Nile virus DNA vaccine induces neutralizing antibody in healthy adults during a phase 1 clinical trial. *The Journal of Infectious Diseases*.

[84] Crotty S., Lohman B. L., Lü F. X., Tang S., Miller C. J., Andino R. (1999). Mucosal immunization of cynomolgus macaques with two serotypes of live poliovirus vectors expressing simian immunodeficiency virus antigens: stimulation of humoral, mucosal, and cellular immunity. *Journal of Virology*.

[85] Wang D., Kandimalla E. R., Yu D., Tang J. X., Agrawal S. (2005). Oral administration of second-generation immunomodulatory oligonucleotides induces mucosal Th1 immune responses and adjuvant activity. *Vaccine*.

[86] Kapadia S. U., Rose J. K., Lamirande E., Vogel L., Subbarao K., Roberts A. (2005). Long-term protection from SARS coronavirus infection conferred by a single immunization with an attenuated VSV-based vaccine. *Virology*.

[87] Bukreyev A., Lamirande E. W., Buchholz U. J., Vogel L. N., Elkins W. R., St Claire M. (2004). Mucosal immunisation of African green monkeys (Cercopithecus aethiops) with an attenuated parainfluenza virus expressing the SARS coronavirus spike protein for the prevention of SARS. *The Lancet*.

[88] Zhi Y., Figueredo J., Kobinger G. P. (2006). Efficacy of severe acute respiratory syndrome vaccine based on a nonhuman primate adenovirus in the presence of immunity against human adenovirus. *Human Gene Therapy*.

[89] Kanesa-thasan N., Smucny J. J., Hoke C. H. (2000). Safety and immunogenicity of NYVAC-JEV and ALVAC-JEV attenuated recombinant Japanese encephalitis virus – poxvirus vaccines in vaccinia-nonimmune and vaccinia-immune humans. *Vaccine*.

[90] DiNapoli J. M., Kotelkin A., Yang L. (2007). Newcastle disease virus, a host range-restricted virus, as a vaccine vector for intranasal immunization against emerging pathogens. *Proceedings of the National Academy of Sciences of the United States of America*.

[91] Bisht H., Roberts A., Vogel L. (2004). Severe acute respiratory syndrome coronavirus spike protein expressed by attenuated vaccinia virus protectively immunizes mice. *Proceedings of the National Academy of Sciences of the United States of America*.

[92] Subbarao K., McAuliffe J., Vogel L. (2004). Prior infection and passive transfer of neutralizing antibody prevent replication of severe acute respiratory syndrome coronavirus in the respiratory tract of mice. *Journal of Virology*.

